# Insulin-Responsive Transcription Factors

**DOI:** 10.3390/biom11121886

**Published:** 2021-12-15

**Authors:** Gerald Thiel, Lisbeth A. Guethlein, Oliver G. Rössler

**Affiliations:** 1 Department of Medical Biochemistry and Molecular Biology, Faculty of Medicine, Saarland University, D-66421 Homburg, Germany; oliver.roessler@uks.eu; 2 Department of Structural Biology and Department of Microbiology & Immunology, Stanford University, Stanford, CA 94305, USA; libbyg@stanford.edu

**Keywords:** ChREBP, Egr-1, Elk-1, FoxO1, liver X receptor, SREBP-1c, USF

## Abstract

The hormone insulin executes its function via binding and activating of the insulin receptor, a receptor tyrosine kinase that is mainly expressed in skeletal muscle, adipocytes, liver, pancreatic β-cells, and in some areas of the central nervous system. Stimulation of the insulin receptor activates intracellular signaling cascades involving the enzymes extracellular signal-regulated protein kinase-1/2 (ERK1/2), phosphatidylinositol 3-kinase, protein kinase B/Akt, and phospholipase Cγ as signal transducers. Insulin receptor stimulation is correlated with multiple physiological and biochemical functions, including glucose transport, glucose homeostasis, food intake, proliferation, glycolysis, and lipogenesis. This review article focuses on the activation of gene transcription as a result of insulin receptor stimulation. Signal transducers such as protein kinases or the GLUT4-induced influx of glucose connect insulin receptor stimulation with transcription. We discuss insulin-responsive transcription factors that respond to insulin receptor activation and generate a transcriptional network executing the metabolic functions of insulin. Importantly, insulin receptor stimulation induces transcription of genes encoding essential enzymes of glycolysis and lipogenesis and inhibits genes encoding essential enzymes of gluconeogenesis. Overall, the activation or inhibition of insulin-responsive transcription factors is an essential aspect of orchestrating a wide range of insulin-induced changes in the biochemistry and physiology of insulin-responsive tissues.

## 1. Introduction: Insulin and the Insulin Receptor

The hormone insulin was discovered 100 years ago, starting scientific exploration of the regulation of metabolic pathways by hormones. Insulin, synthesized and secreted by pancreatic β-cells, is the key hormone for regulating glucose homeostasis. It lowers hepatic gluconeogenesis and stimulates glucose uptake into adipocytes and skeletal muscle. A high glucose concentration in the blood is an important trigger for insulin release. Moreover, ligands of G protein-coupled receptors and receptor tyrosine kinases induce insulin exocytosis. Insulin binds to the insulin receptor that is found in skeletal muscle, adipocytes, hepatocytes, pancreatic β-cells, and several areas of the central nervous system. The insulin receptor is a receptor tyrosine kinase, which is formed by a tetramer with two α and two β subunits. The two subunits of the insulin receptor are derived from a single precursor molecule. Insulin binds to the α-subunit which is connected via a disulfide linkage to the transmembrane β-subunit. Insulin binding activates the enzymatic function of the receptor, leading to a transphosphorylation and a further increase in kinase activity, allowing the tyrosine phosphorylation of several insulin receptor substrates that are connected with various signaling cascades within the cell.

Many biological functions have been attributed to insulin receptor signaling. Insulin regulates the transport of glucose into the cells via insulin-responsive GLUT4 transporters, the regulation of growth and proliferation, the control of glucose homeostasis, and the regulation of food intake [1,2,3]. Insulin controls transcription of genes encoding proteins involved in the regulation of glycolysis, gluconeogenesis, lipogenesis, and food intake. This review summarizes available data concerning insulin-responsive transcription factors that contribute to the effects of insulin in insulin-responsive tissues. The article outlines how the activity of several transcription factors executes the function of insulin in regulating many cellular activities, including cell growth, lipogenesis, and homeostasis.

## 2. Insulin Receptor-Induced Intracellular Signaling Cascades

**Figure 1:**
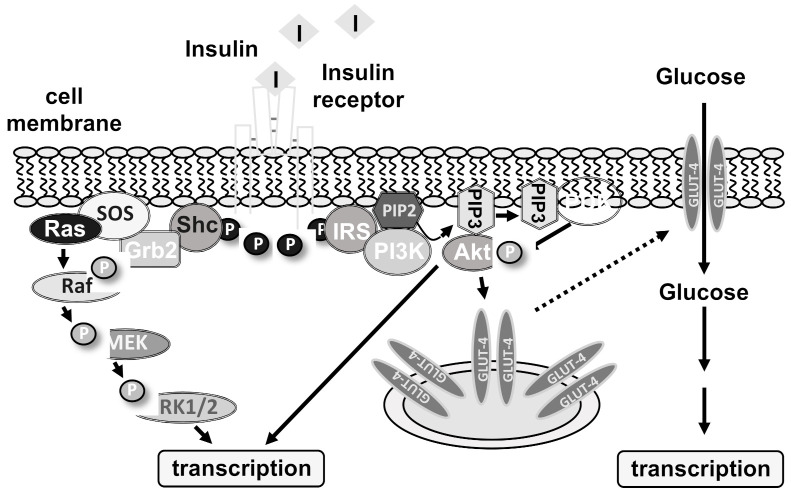
Insulin receptor signaling. This figure shows a schematic model of two kinase cascades activated by insulin receptor stimulation. Insulin binding to the insulin receptor triggers tyrosine phosphorylation of the receptor and the adapter proteins Shc and insulin receptor substrates (IRS). Shc binds to the adaptor protein Grb2 that interacts with the guanine nucleotide exchange factor SOS (son of sevenless), generating the activated GTP-bound protein Ras. This G protein binds and activates the protein kinase Raf that in turn phosphorylates the protein kinase MEK (Mitogen-activated protein kinase/extracellular signal-regulated protein kinase kinase). Finally, MEK phosphorylates and activates ERK1/2 (extracellular signal-regulated protein kinase). The second cascade involves the lipid kinase phosphatidylinositol 3-kinase (PI3 kinase) which binds with its p85 regulatory domain to phosphorylated IRS. This activates the catalytic subunit of PI3 kinase, which catalyzes the phosphorylation of phosphatidylinositol-4,5-bisphosphate (PIP2) to phosphatidylinositol-3,4,5-trisphosphate (PIP3). This phospholipid binds to the protein kinase B/Akt and 3-phosphoinositide-dependent protein kinase (PDK) and triggers their activation. The protein kinases ERK1/2 and protein kinase B/Akt function as signal transducers to transport the hormonal information into the nucleus. Transcriptional regulators are phosphorylated, leading to alterations in gene transcription. Insulin receptor activation additionally triggers the incorporation of GLUT4 transporters into the plasma membrane, leading to an influx of glucose into adipocytes and skeletal muscle cells. The rise in glucose functions as a signal to further activate a transcriptional response.

The insulin receptor is a receptor tyrosine kinase having an extracellular ligand binding domain, a single transmembrane domain, and a cytoplasmic domain containing the tyrosine kinase function. Insulin binding to the receptor α-subunit activates its enzymatic function, leading to a transphosphorylation of the β-subunit of the receptor. Moreover, several insulin receptor substrates are phosphorylated on tyrosine residues. As a result, intracellular signaling cascades are activated that include the enzymes extracellular signal-regulated protein kinase (ERK1/2), phosphatidylinositol 3-kinase (PI3 kinase), protein kinase B/Akt, and phospholipase Cγ (Figure 1). Activation of phospholipase Cγ induces a rise in intracellular Ca^2+^ via IP_3_ and the IP_3_ receptor that leads to a further activation of ERK1/2 via protein kinase C. Insulin receptor stimulation additionally triggers the translocation of GLUT4 transporters into the plasma membrane which leads to an influx of glucose in the cells. The rise in glucose functions as a trigger for inducing transcriptional changes.

## 3. Transcription Factors under Control of Insulin Receptor Signaling

### 3.1. The Elk-1-Egr-1 Axis

**Figure 2:**
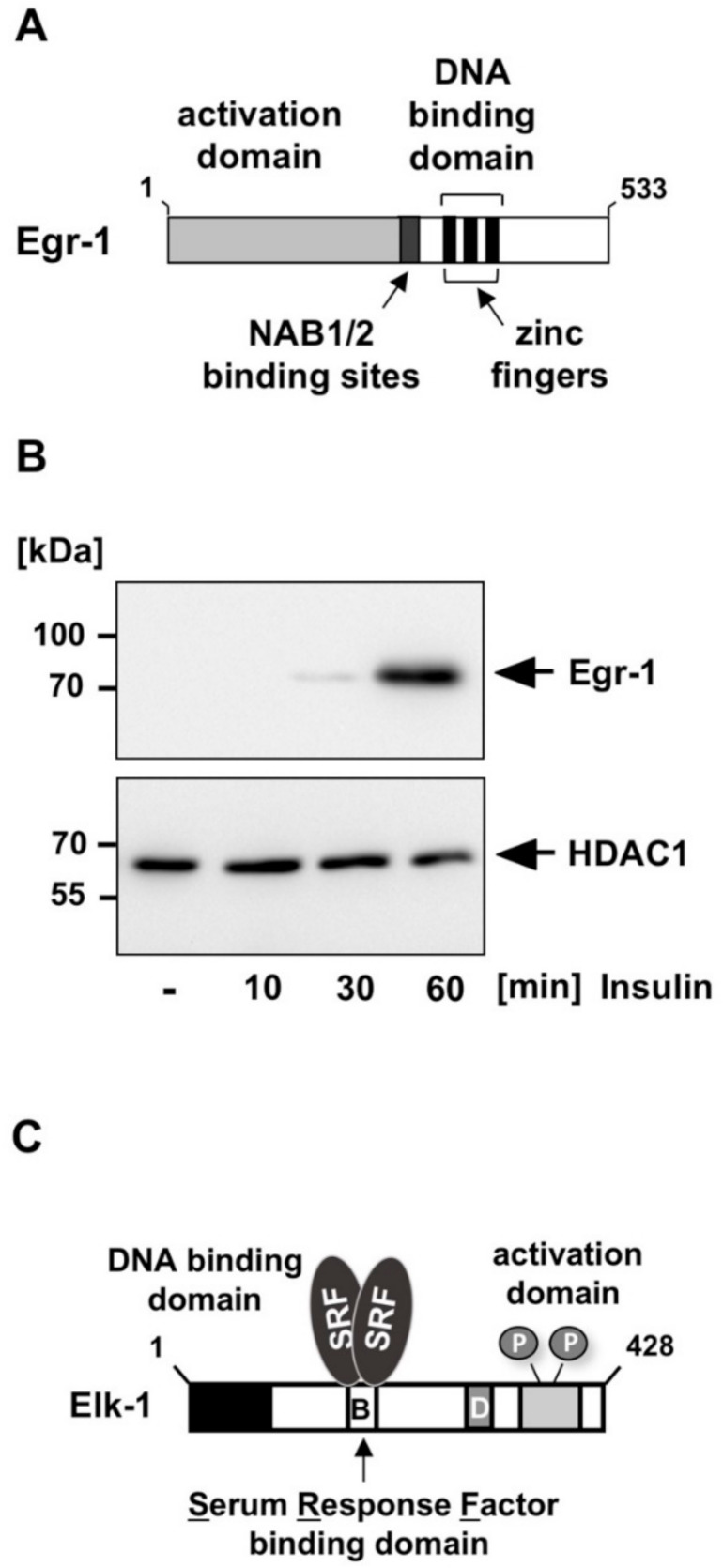
Insulin receptor stimulation activates the biosynthesis of the zinc finger transcription factor Egr-1 under the control of Elk-1. (**A**) Modular structure of Egr-1. The protein has an extended N-terminal activation domain and a C-terminal DNA binding domain with three zinc finger motifs as DNA interaction domain. In addition, there is a binding site for the transcriptional co-repressor proteins NAB1 and NAB2. (**B**) Insulin treatment of insulin receptor-expressing HIRcB cells induced the biosynthesis of Egr-1. HIRcB cells were cultured for 24 h in medium containing 0.05% serum and then stimulated with insulin (100 nM). Nuclear extracts were prepared and subjected to Western blot analysis. The blot was developed with an antibody directed against Egr-1. An antibody detecting histone deacetylase-1 (HDAC1) immunoreactivity was used as a loading control. Reproduced with modifications from ref. [7] with permission from Elsevier. (**C**) Modular structure of Elk-1. The protein has an N-terminal DNA binding domain and a C-terminal activation domain. The B domain is the interaction domain with serum response factor. The domain D is the interaction site for ERK1/2 and c-Jun-*N*-terminal protein kinase.

Egr-1 (early growth response-1) is a transcription factor that interacts with DNA via three zinc finger motifs (Figure 2A). Egr-1 function is regulated via its biosynthesis. Expression of Egr-1 is not detectable in resting cells. However, stimulation of insulin receptor-expressing fibroblasts with insulin induces the biosynthesis of Egr-1 (Figure 2B) [4,5,6,7]. The biosynthesis of Egr-1 is also induced following stimulation of other receptor tyrosine kinases such as the epidermal growth factor (EGF) receptor or the BDNF-responsive tyrosine receptor kinase B (TrkB) [8,9,10], indicating that receptor tyrosine kinase signaling converges to the activation of the Egr-1 encoding gene. Receptor tyrosine kinases use the protein kinase ERK1/2 as signal transducer for activating Egr-1 biosynthesis. Previous studies have shown that ERK1/2 functions as a strong inducer of Egr-1 biosynthesis [9,10,11,12]. Accordingly, upregulation of Egr-1 promoter activity by insulin was attenuated in the presence of MAP kinase phosphatase-1 [7], an enzyme that catalyzes the dephosphorylation and inactivation of ERK1/2 in the nucleus. In contrast, activation of PI3 kinase/Akt is not involved in insulin-induced activation of Egr-1 expression [5].

Egr-1 is known to regulate cellular growth and proliferation, as shown for astrocytes, hepatocytes, keratinocytes, and pancreatic β-cells [8,9,10,13,14]. Insulin as a potent anabolic hormone enhances cellular growth and proliferation. Accordingly, mutations of the insulin receptor result in growth retardation in humans [15]. Delayed hepatocellular mitotic progression and an impairment of liver regeneration has been reported for insulin receptor-expressing hepatocytes in Egr-1-deficient mice [13]. Thus, growth of hepatocytes requires insulin receptor-induced Egr-1 expression. The insulin receptor is also expressed in pancreatic β-cells. β-cell-specific expression of a dominant-negative mutant of Egr-1, which blocks the transcription of Egr-1 target genes, reduced the size of pancreatic islets by 20% [14], indicating that stimulated insulin receptors are essential for generating islets of normal size via activating Egr-1 expression and Egr-1 target gene transcription. One of the identified target genes of Egr-1 is the gene encoding basic fibroblast growth factor [14,16] known to stimulate DNA synthesis and proliferation. Another target gene of Egr-1 encodes pancreatic duodenum homeobox-1 protein (Pdx-1) [17], a major regulator of insulin gene transcription. Accordingly, inhibition of Egr-1 activity in β-cells of transgenic mice resulted in reduced Pdx-1 expression, and insulin expression [14]. 

Expression of Egr-1 is mainly regulated by Elk-1, a ternary complex factor that binds, together with the serum response factor, to the serum response element (SRE) [18] (Figure 2C). The Egr-1 promoter contains five copies of the SRE, which function as insulin-responsive elements [7]. Elk-1 contains a phosphorylation-responsive activation domain and the protein is phosphorylated by ERK1/2 and other MAP kinases. The insulin-induced stimulation of the Egr-1 promoter is almost completely blocked in insulin-stimulated HIRcB fibroblasts in the presence of a dominant-negative mutant of Elk-1, indicating that Elk-1 controls Egr-1 expression. The importance of Elk-1 in insulin-induced gene transcription is supported by the observation that the transcriptional activation potential of Elk-1 is increased in HIRcB cells following stimulation of the insulin receptor [7]. Thus, insulin receptor stimulation results in the expression of activated Elk-1 in the cells. 

Elk-1 regulates transcription of genes containing one or more SREs in their regulatory regions. Additionally, Elk-1 is a regulator of the transcription factor activator protein-1 (AP-1) [19,20,21,22,23], which is composed of basic region-leucine zipper proteins of the c-Jun, c-Fos, and activating transcription factor (ATF) family of transcription factors. The c-Fos protein is found in many AP-1 complexes and AP-1 activity is regulated by Elk-1 via the control of c-Fos expression. Accordingly, expression of a dominant-negative mutant of Elk-1 attenuated the upregulation of AP-1 activity in insulin-stimulated fibroblasts [7], shedding light to the important role of Elk-1 in insulin-induced transcriptional regulation by inducing SRE, Egr-1, and AP-1-controlled gene transcription.

Elk-1 is important for regulating proliferation and apoptosis in astrocytes, fibroblasts, and pancreatic β-cells [18]. Experiments involving a dominant-negative mutant of Elk-1 revealed that Elk-1 and related ternary complex factors are required to induce proliferation [10]. The analysis of two insulin receptor tyrosine kinase mutants (R1174N, P1178L) showed that no insulin-induced activation of Elk-1 and no mitogenic response was measured and the phosphorylation and activation of ERK1/2 was greatly reduced. In contrast, stimulation of the insulin receptor still induced IRS-1 phosphorylation and PI3 kinase activation [24]. These data indicate that insulin receptor-induced proliferation requires an activation of ERK1/2 and Elk-1 and is independent of PI3 kinase activation. Elk-1 in turn activates Egr-1 to continue the mitogenic signaling cascade. In pancreatic β-cells, impairment of Elk-1 induced transcription leads to the generation of significantly smaller islets [25], due to an increased rate of apoptosis. Thus, insulin contributes to proliferative responses of pancreatic β-cells by a sequential activation of ERK1/2, Elk-1, and Egr-1. In hepatocytes, it has been shown that a lack of the AP-1 constituting transcription factor c-Jun induces cell death [26], suggesting that the survival of the cells is induced by insulin-triggered activation of AP-1.

Moreover, activation of Elk-1 and Egr-1 in pancreatic β-cells is required for the regulation of glucose homeostasis [14,25]. In addition, the transcriptional activity of AP-1 is essential in pancreatic β-cells for the regulation of glucose homeostasis [27], indicating that insulin receptor signaling in pancreatic β-cells directly supports the maintenance of glucose homeostasis via activating Elk-1, Egr-1, and AP-1. Morphometric analysis of transgenic mice expressing dominant-negative mutants of either Egr-1 or Elk-1 in pancreatic β-cells revealed that the islets were significantly smaller when the transgene was expressed [14,25], indicating that Egr-1 and Elk-1 induce a gene expression program that is required for the generation of islets of adequate size. These data imply that impaired glucose tolerance observed in transgenic mice expressing dominant-negative mutants of either Egr-1 or Elk-1 in pancreatic β-cells is the consequence of β-cell loss. In contrast, genetic inhibition of AP-1 activity in β-cells had no effect on the size of pancreatic islets [27], indicating that impaired glucose tolerance of transgenic mice expressing a dominant-negative mutant of AP-1 in pancreatic β-cells is not due to a loss of β-cells.

Insulin signaling also plays an important role in the regulation of adipogenesis through activation of Elk-1 via the ERK1/2 signaling pathway. Elk-1, in turn, activates expression of Krox20, a transcription factor related to Egr-1, that is highly expressed in adipocytes. Elk-1 is controlled by phosphorylation and by the subsequent binding of the Mediator subunit Med23 to phosphorylated Elk-1. Accordingly, adipogenesis was blocked in Med23 and Elk-1-deficient cells [28]. 

### 3.2. Upstream Stimulatory Factor (USF)

**Figure 3:**
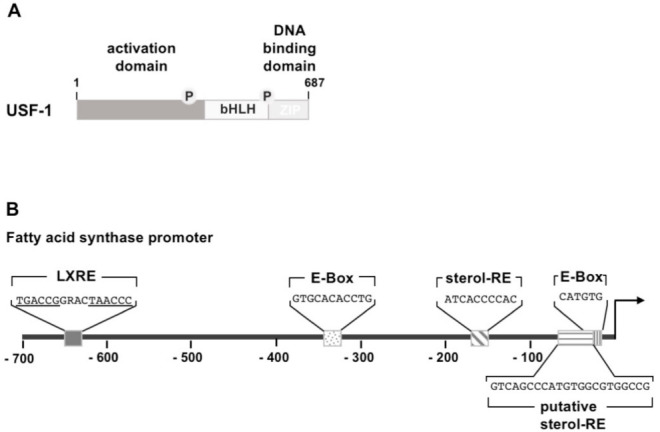
Modular structure and binding sites of upstream stimulatory factor (USF). (**A**) The USF protein USF-1 and USF-2 have a similar domain structure, encompassing an N-terminal activation domain, a basic helix-loop-helix domain (bHLH), and a C-terminal leucine zipper (LZ). (**B**) Transcription factor binding sites within the proximal fatty acid synthase promoter. Two USF binding sites (E-Box) are depicted. In addition, there is a binding site for SREBP-1c (sterol-responsive element). A second tandem binding site for SREBP-1c, overlapping with the proximal E-box, has been suggested. A binding site for liver X receptor (LXRE) is found in the proximal fatty acid synthase promoter. Additionally, there is an upstream binding site for carbohydrate response element-binding protein (ChREBP) at position -7382 to -6970, which is not depicted in the cartoon. The fatty acid synthase promoter sequence was obtained from GeneBank X54671.1.

Insulin stimulates transcription mediated by upstream stimulatory factors USF-1 and USF-2 which belong to the group of basic helix-loop-helix leucine zipper (bHLH-LZ) transcription factors (Figure 3A). The HLH domain consists of two α-helices, connected by a loop sequence. The basic domain, used for DNA binding, is found within the first helix, allowing interaction with a DNA motif known as E-box, encompassing the sequence 5′-CANNTG-3′, as either as homodimer or as an USF-1/USF-2 heterodimer. Structural analysis revealed that USF may also function as a bivalent homotetramer [29] that is able to interact simultaneously with two spatially separated E-boxes. The second helix and the leucine zipper facilitate interaction with other bHLH-LZ proteins. USF forms, for example, a complex with the insulin-responsive transcription factor sterol regulatory element-binding protein-1c (SREBP-1c), resulting in a synergistic activation of lipogenic genes [30]. The transcriptional activation domain is found on the N-terminus of the USF protein. 

USF is a regulator of fatty acid synthase gene transcription. The encoded enzyme, a key enzyme of lipogenesis, catalyzes the conversion of acetyl-CoA and malonyl-CoA to palmitate. The fatty acid synthase gene is controlled in lipogenic tissues by insulin and nutrients. USF binds to two sites within the fatty acid synthase promoter (Figure 3B) and both sites are necessary for transcriptional activation in vivo. Mutational inactivation of the USF binding sites abolished feeding or insulin-induced activation of the fatty acid promoter [31]. In addition, the insulin-inducible transcription factor SREBP-1c binds to the fatty acid synthase gene promoter, encompassing the consensus site 5′-ATCACCCCAC-3′ (sterol-responsive element), but needs the presence of the proximal E-box. The synergistic activation of fatty acid synthase expression by USF and SREBP requires the interaction of both proteins.

Likewise, other genes encoding enzymes required for lipogenesis, including the genes encoding acetyl-CoA carboxylase, ATP-citrate lyase, and mitochondrial glycerol-3-phosphate acyltransferase are responsive to insulin/nutrient stimulation and contain E-boxes and SREBP-1c binding sites in their regulatory region [32], suggesting that the close presence of E-boxes and sterol response elements are a common theme in lipogenic gene regulation. The importance of USF in regulating lipogenic gene transcription is underlined by the fact that fatty acid synthase gene expression is significantly impaired in USF-deficient mice [33].

Chromatin immunoprecipitation experiments showed that USF is bound to the E-boxes of the fatty acid synthase gene and the mitochondrial glycerol-3-phosphate acyltransferase gene in both resting and stimulated cells [31,34], indicating that insulin does not stimulate DNA binding of USF. Rather, the activity of USF is controlled via posttranslational modifications, involving insulin-stimulated phosphorylation by the DNA-dependent protein kinase and acetylation by the acetyltransferase P/CAF. Under resting conditions, USF is deacetylated, catalyzed by the histone deacetylase HDAC9 [34,35]. HDAC9 binds in the liver to the fatty acid synthase promoter and the mitochondrial glycerol-3-phosphate acyltransferase promoter only in fasted, but not in fed conditions, leading to a deacetylation of USF and the repression of transcription. Thus, HDAC9 is a corepressor of lipogenic genes. Upon feeding or insulin stimulation, protein phosphatase PP1 translocates into the nucleus and dephosphorylates the protein kinase DNA-PK, which is activated by dephosphorylation. DNA-PK phosphorylates USF-1 and allows the recruitment of the acetyltransferase P/CAF which catalyzes the acetylation of USF-1 and the subsequent transcription of the fatty acid synthase gene. Thus, there is a switch-like mechanism involving phosphorylation and acetylation and dephosphorylation and deacetylation to regulate USF-mediated transcription of lipogenic genes according to the nutritional status and the insulin concentration [34].

### 3.3. Sterol Regulatory Element-Binding Protein-1c (SREBP-1c)

Insulin induces expression of SREBP-1c [36], a transcription factor belonging to the basic-helix-loop-helix leucine zipper (bHLH-LZ) family of transcription factors that is essentially involved in the regulation of lipogenic genes. The related SREBP-2 protein, in contrast, is a regulator of genes encoding proteins of sterol biosynthesis. All SREBP proteins bind to a genetic element known as the sterol-response element, encompassing the classic sequence 5′-ATCACCCCAC-3′ or variations of this sequence termed sterol-response element-like sites [37].

Insulin-induced expression of fatty acid synthase has been shown to be regulated by SREBP-1c [38]. Moreover, SREBP1-c has been identified as a transcriptional regulator of lipid synthetic genes [39]. Expression of lipogenic genes was reduced or completely abolished in the liver lacking SREBP-1c [40]. Figure 3B shows that there is a consensus SREBP-1c binding site within the fatty acid synthase gene promoter and it has been shown that SREBP-1c activates transcription of this gene together with USF [31]. It has been suggested that SREBP-1c additionally interacts directly with a proximal region of the fatty acid synthase gene promoter, encompassing the proximal E-box (Figure 3B). However, chromatin immunoprecipitation experiments performed with cells derived from the liver of transgenic mice containing a reporter gene under the control of 131 nucleotides of the fatty acid synthase promoter showed that only USF bound in vivo to the proximal E-box and not SREBP. Interestingly, mutation of the proximal E-box prevented binding of SREBP to the sterol-responsive element within the fatty acid promoter in vivo, indicating that an interaction between SREPB and USF is required for the stimulation of fatty acid synthase gene transcription [31].

In addition to the fatty acid synthase gene, there are functional SREBP binding sites in the regulatory region of the acetyl-CoA carboxylase gene and other lipogenic genes [32]. SREBP-1c has been identified as a mediator connecting insulin stimulation with glucokinase gene expression in the liver [41]. Glucokinase expression in the liver relies on the stimulation of the cells with insulin. Thus, SREBP-1c-induced expression of glucokinase provides the synthesis of metabolites and energy required for lipid biosynthesis.

Activation of cAMP-dependent protein kinase phosphorylates SREBP-1c on serine residue S314, resulting in attenuation of SREBP-1c DNA binding and target gene transactivation [42]. cAMP-dependent protein kinase additionally suppresses SREBP expression by phosphorylation of liver X receptor, a major regulator of the SREBP-1 gene [43]. Moreover, phosphorylation of SREBP-1c by glycogen synthase kinase-3 (GSK3) creates a binding site for the ubiquitin ligase Fbw7 that triggers ubiquitination and proteolysis of SREBP-1c [44]. Insulin inhibits GSK3 via the activation of Akt, indicating that the degradation of SREBP-1c occurs in the absence of insulin.

SREBP-1c activity is under epigenetic control. Insulin and high glucose concentration trigger an acetylation of SREBP-1c on arginine residues K289 and K309, involving the acetyltransferase protein p300, while deacetylation, catalyzed by SIRT-1, impairs binding of SREBP-1c to its target genes [44,45]. SIRT1, the human homologue of the yeast Sir2 protein (silencing information regulator 2), is as a NAD^+^-dependent deacetylase that functions as a sensor for NAD^+^. SIRT1 deacetylates acetylated proteins including histones and various transcription factors such as SREBP-1c. SIRT1 is activated by glucagon and the rise in intracellular cAMP and is as a positive regulator of gluconeogenesis and fatty acid oxidation [46].

**Figure 4:**
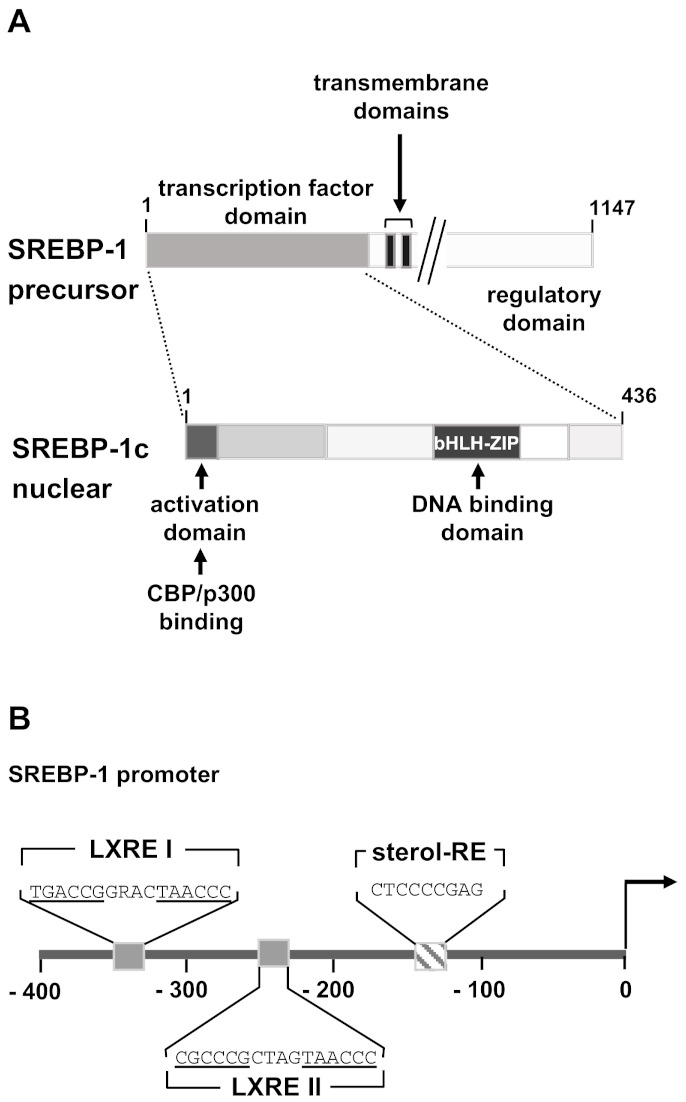
Modular structure and promoter sequence of SREBP-1. (**A**) The SREBP-1 precursor protein is an ER-associated membrane protein consisting of an N-terminal transcription factor domain, two transmembrane regions, and a regulatory domain that interacts with the ER protein SCAP. The precursor protein is cleaved by two proteases, releasing the functional SREBP-1c protein. SREBP-1c has a short N-terminal activation domain that contains binding sites for transcriptional coactivator proteins. Next to the activation domain is a domain rich in proline and serine residues. The basic region helix-loop-helix-leucine zipper (bHLH-Zip) domain is found on the C-terminus of the molecule. (**B**) Transcription factor binding sites within the murine SREBP-1 gene promoter. Two binding sites for liver X receptor (LXRE1 and LXRE2) and a binding site for SREBP-1c (sterol-RE) are depicted. The sequence was obtained from GenBank (AB046200.1).

The activation of SREBP-1c is complex and involves trafficking between the endoplasmic reticulum, the Golgi, and the nucleus, regulated by numerous proteins and signals [47]. SREBP proteins are expressed as precursor proteins inserted in the ER membrane. An insulin-induced processing cascade of the SREBP-1 precursor is required to generate mature SREBP-1. The transcriptional activity of SREBP-1c depends on the concentration of mature SREBP-1c in the nucleus. The precursor protein is embedded in the membrane of the endoplasmic reticulum via two transmembrane regions. Both the N- and C-terminal ends are facing the cytoplasm. The N-terminal domain contains the transactivating domain and the bHLH-LZ domain as shown in Figure 4A. The C-terminal regulatory domain binds under basal conditions to the SREBP cleavage-activating protein (SCAP) in the ER membrane. In addition, the SREBP precursor-SCAP complex interacts with insulin-induced gene proteins (INSIGs), immobilizing the SREBP precursor in the ER membrane. Insulin receptor stimulation activates protein kinase B/Akt, leading to degradation of the INSIG proteins. Likewise, high concentrations of cellular sterols induce the degradation of INSIGs. As a result, the SREBP precursor-SCAP complex is transported to the Golgi, where the SREBP precursor is cleaved by the S1P and S2P proteases. Insulin additionally activates SREBP processing by stimulating the serine/threonine kinase mTORC1 (mammalian target of rapamycin complex 1) which is a major effector protein downstream of Akt [35,48].

Insulin stimulation activates expression of the SREBP-1 precursor protein and additionally enhances the maturation of the precursor protein to transcriptionally active SREBP-1c involving insulin-induced PI3 kinase/Akt [38,41,49,50]. Insulin-induced expression of SREBP-1c is regulated by the insulin-responsive transcription factor liver X receptor [35,51]. Moreover, once activated SREBP-1c binds to its own promoter and activates SREBP expression. Binding sites for liver X receptor and SREBP-1c are found in the SREBP-1 promoter (Figure 4B).

SREBP-1c has been proposed as a negative regulator of phosphoenolpyruvate carboxykinase (PEPCK) gene transcription in the liver. PEPCK is a key enzyme of gluconeogenesis, catalyzing the generation of phosphoenolpyruvate from oxalacetate. PEPCK expression is high under fasting conditions and major activators are cAMP, PKA, and CREB. In contrast, insulin stimulation shuts down PEPCK expression. There are two proposed sterol response elements in the PEPCK promoter that bind SREBP with low affinity. One of them encompasses the sequence −590 5′-ATCACCCCTC-3′-581 [52], which contains only one mismatch in comparison to the classic sequence 5′-ATCACCCCAC-3′ found in the fatty acid synthase gene promoter. Expression of SREBP-1c in HepG2 hepatoma cells reduced transcription of a PEKCK promoter/reporter gene by 70% suggesting that SREBP-1c functions as a repressor of PEPCK gene transcription. It has been suggested that SREBP-1c competes with Sp1, which binds to a DNA site overlapping with the proposed SREBP-1c binding site. The expression of PEPCK is upregulated by FoxO1 and insulin administration has been shown to reduce binding of FoxO1 and its co-activator PCG-1α to the PEPCK gene in vivo [53].

### 3.4. Carbohydrate Response Element-Binding Protein (ChREBP)

**Figure 5:**
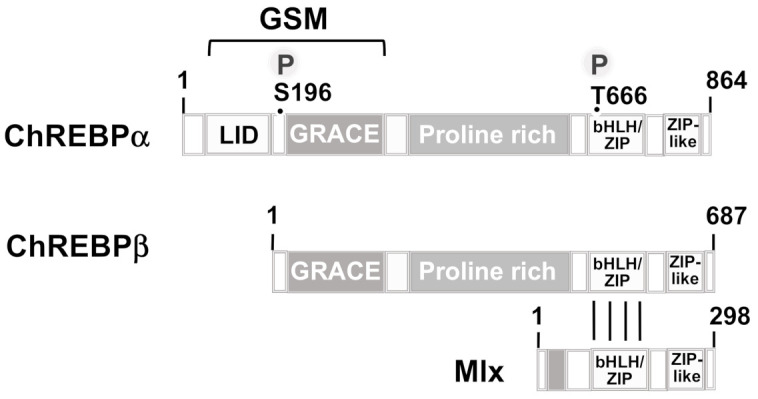
Modular structure of ChREBPα, ChREBPβ, and Mlx. The ChREBP proteins contain a C-terminal helix-loop-helix/zipper domain (bHLH-ZIP) that is used for binding to DNA and for dimerization with Mlx. The C-termini contain a leucine zipper-like domain (ZIP-like). The N-terminal glucose-sensing module (GSM), composed of a glucose-inhibitory domain (LID) and a glucose-response activation conserved element (GRACE), is depicted along with two phosphorylation sites for cAMP-dependent protein kinase.

SREBP-1c has been regarded as the principal activator of lipogenesis at the transcriptional levels. However, fatty acid synthesis was only reduced by 50% in SREBP-1c-deficient mice [40], suggesting that there must be an additional protein that regulates glucose and insulin-induced activation of lipogenesis. A major function of insulin is the stimulation of glucose uptake into adipocytes and skeletal and cardiac muscle cells executed by the translocation of GLUT4 into the plasma membrane [54]. This influx of glucose into the cells functions as a trigger for activating carbohydrate response-element binding protein (ChREBP), the dominant lipogenic transcription factor highly expressed in adipocytes and hepatocytes [55,56,57,58]. Thus, insulin does not activate ChREBP directly, but rather plays a permissive role in stimulating glucose uptake into the cells. There are two isoforms of ChREBP, ChREBPα, and ChREBPβ (Figure 5). ChREBPβ is transcribed from an alternative promoter of the ChREBP gene. ChREBP is a bHLH-ZIP transcription factor that dimerizes with Mlx (Max-like protein X) and binds to the carbohydrate response-element (ChoRE) as a heterotetramer [59]. The importance of the dimerization partner Mlx has been elucidated in experiments involving a dominant-negative Mlx mutant (dnMlx), containing mutations of two basic residues within the basic domain. The mutant was able to heterodimerize with ChREBP, because the HLH-LZ domain was intact. However, DNA binding of the ChREBP-dnMlx was attenuated. As a result, glucose-induced upregulation of lipogenic gene transcription was inhibited [60]. The ChREBP-Mlx palindromic binding site was determined to be 5′-CAYGNGNNNNNCNCRTG-3′ (Y = C or T; R = A or G) [59]. The spacing of 5 base pairs between both palindromes is important, as a spacing of 6 base pairs significantly reduces glucose responsiveness, whereas a spacing of 4 base pairs between both E-boxes results in a genetic element that can no longer provide glucose responsiveness [61].

Under basal conditions, ChREBP is a cytosolic phosphoprotein that functions as a substrate for cAMP-dependent protein kinase. Phosphorylation by PKA and dephosphorylation by protein phosphatase 2A has been suggested to regulate the intracellular location of ChREBP and thus its biological activity. However, this model was not generally accepted and experimental data, involving mutations of the S196 phosphorylation site, did not support it. Importantly, 14-3-3 proteins bind to the N-terminal region and immobilize the protein in the cytoplasm, suggesting that the ChREBP-14-3-3 complex has to be broken up to allow nuclear translocation of ChREBP. The ChREBPα molecule contains a glucose-sensing module (GSM) in its N-terminal region that encompasses a low-glucose inhibitory domain (LID) and a glucose-response activation conserved element (GRACE) (Figure 5). The ChREBPβ isoform is a shorter variant in comparison to ChREBPα, lacking the N-terminal 177 amino acids, i.e., most of the LID domain. The function of this isoform is not yet determined and a feed-forward mechanism, involving the regulation of ChREBPβ expression by ChREBPα or even by ChREBPβ has been proposed [62,63] as well as a role for ChREBPβ in a negative feedback loop [58]. It has been suggested that there is an intramolecular binding of the LID domain to the GRACE domain under low glucose conditions, leading to an inhibition of ChREBPa activity. Binding of glucose metabolites (glucose-6-phosphate, xylulose 5-phosphate, fructose-2,6-bisphosphate) disrupts this interaction and activates ChREBPα [58]. Accordingly, a ChREBPα mutant that lacks the N-terminal 196 amino acids and thus most of the LID domain, is constitutively active. Acetylation of ChREBP on lysine residue 672 by the acetyltransferase p300 further stimulates the transcriptional activity [64]. ChREBP is negatively regulated by a number of metabolites, including saturated and non-saturated branched chain α-ketoacids, or AMP that all target nucleo-cytoplasmic trafficking [65,66]. Interestingly, the fact that ketone bodies function as allosteric inhibitors of ChREBP nuclear translocation suggests that ketogenic diets may be useful for treating diabetes by inhibiting transcriptional regulation via ChREBP and subsequent de novo lipogenesis.

ChREBP regulates together with SREBP-1c the genetic program of lipogenesis. A ChoRE has been identified in various lipogenic genes encoding acetyl-CoA carboxylase, fatty acid synthase, and stearoyl-CoA desaturase-1 [67]. The ChoRE within the fatty acid synthase promoter is further upstream in comparison to the binding sites for USF, SREBP, and liver X receptor and encompasses the sequence 5′-CATGTG*CCACA*GGCGTG-3′ (-7214/-7198) [68]. The ChoRE of the acetyl-CoA carboxylase gene is within the proximal promoter region (sequence 5′-CATGTG*AAAAC*ACTGTG-3′).

Moreover, ChREBP regulates the expression of genes encoding enzymes involved in the regulation of glycolysis and the pentose phosphate pathway, thus providing essential metabolites (acetyl-CoA) and reductive power (NADPH) essential for lipogenesis [57,67,69]. A functional ChoRE has been identified in the ChREBPβ promoter [63]. In addition, ChREBP activates the transcription of SREBP-1, and thus leads to the expression of a transcription factor that is involved in the response to nutrients and insulin [70]. The important role of ChREBP in the regulation of lipogenesis has been proven in downregulation experiments, showing that attenuation of ChREBP expression resulted in systemic insulin resistance [71]. ChREBP-deficient mice showed a 60% reduction in lipogenesis [69]. A microarray analysis involving a dominant-negative Mlx mutant identified many lipogenic genes within the entire pathway of lipogenesis as targets of ChREBP-Mlx [59]. Moreover, expression of a constitutively active mutant of ChREBP in insulinoma cells greatly induced lipid droplet accumulation [72]. ChREBP has also been identified as a regulator of cell proliferation in hematopoietic and pancreatic β-cells [56].

### 3.5. Liver X Receptor

**Figure 6:**
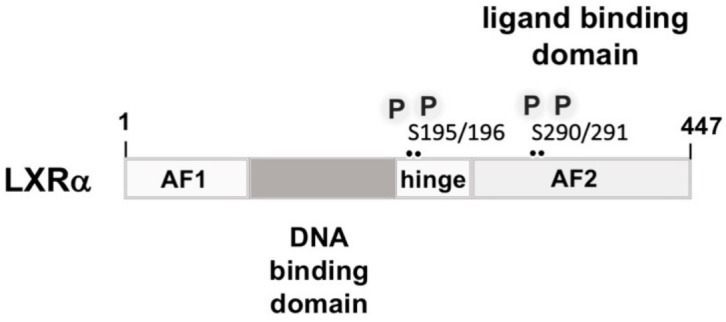
Modular structure of and binding sites of liver X receptor. Liver X receptor α (LXRα) has the common modular structure of nuclear steroid receptors, containing an N-terminal region with a ligand-independent transcriptional activation function (AF1), a DNA binding domain encompassing two zinc fingers, and a C-terminal ligand-binding domain that is separated from the DNA binding domain by a hinge region. The ligand-binding domain is additionally important for dimerization and transcriptional activation, containing the AF2 transcriptional activation domain. The PKA phosphorylation sites “P” are depicted.

Insulin stimulates expression of liver X receptor α (LXRα) in primary hepatocytes by increasing the steady-state mRNA levels of LXRα and by increasing the half-life of LXRα transcripts [36]. However, the authors of this study did not directly measure LXRα transcriptional activity. Other investigators proposed that insulin may increase the activity of LXRα by either stimulating the biosynthesis of a ligand for LXRα or by increasing the activity of a transcriptional coactivator [73]. LXRα is a nuclear hormone receptor that shows the typical modular structure of steroid receptors (Figure 6). The N-terminal region contains the AF1 ligand-independent transcriptional activation domain. The DNA binding domain is characterized by the presence of two zinc finger motifs. A hinge region separates the DNA binding domain from the C-terminal domain that is responsible for ligand binding, coregulator binding dimerization, and transcriptional activation via the ligand-dependent AF2 transcriptional activation domain. There are two LXR isoforms, LXRα and LXRβ, with an abundant expression of the LXRα isoform in lipogenic tissue. The natural ligands for LXRs are cholesterol-derived oxysterols. In the absence of the ligand LXR interacts with co-repressor proteins such as the nuclear receptor co-repressor (NCoR) which recruits histone deacetylase enzymes to the transcription unit. Binding of the ligand induces a conformation change, resulting in the dissociation of the repressor proteins and the recruitment of transcriptional co-activators. Both LXR proteins bind together with their partner, the 9-cis retinoic acid receptor, to the LXR response element (LXRE), encompassing the sequence 5′-AGGTCA*NNNN*AGGTCA-3′. This cognate site consists of two direct repeats of the sequence AGGTCA that are separated by four nucleotides.

LXRα is involved in the control of insulin-mediated lipogenesis by directly activating gene transcription of lipogenic genes, including genes encoding fatty acid synthase (Figure 3B), acetyl CoA carboxylase, and stearoyl CoA desaturase [74]. Accordingly, insulin-induced expression of lipogenic genes was reduced or completely abolished in the LXRα and LXRα/β-deficient mice [36,75].

Additionally, LXRα indirectly activates lipogenesis by stimulating SREBP-1c expression [76]. Two binding sites for LXR have been identified in the SREBP-1c promoter (Figure 4B) and data have been published that indicate that insulin activates SREBP-1 promoter activity mainly by upregulating LXRα activity [73]. Therefore, reduced levels of SREBP-1c and lipogenic enzymes have been measured in LXRα/LXRβ-deficient mice [36]. Experiments involving SREBP-1c-deficient mice underlined the importance of SREBP-1c for liver X receptor-induced activation of lipogenic genes [40].

However, stimulation of SREBP-1c deficient mice with a synthetic LXR agonist still results in a stimulation of lipogenic genes [40], suggesting that LXR can activate lipogenesis independently of SREBP-1. Two functional LXRE have been identified in the promoter of the ChREBP gene, encompassing the sequence 5′-CGGGTA*CTAG*AGGGCAGGCGAGAAAGGCAA*TGAG*AGGTGA-3′ (-2432 to -2393), and it was shown that this element mediates the upregulation of ChREBP expression via LXR [77]. Thus, LXR regulates lipogenic and glycolytic gene transcription via activating both SREBP and ChREBP expression.

Under fasting conditions, phosphorylation of LXR by PKA on serine residues within the ligand binding domain impairs dimerization with RXR and DNA binding to the LXRE. In addition, recruitment of co-activators is inhibited, and recruitment of co-repressors is enhanced [43]. Additionally, LXRα stimulates its own expression mediated by three LXRα binding sites in the LXRα gene.

### 3.6. FoxO1

**Figure 7:**
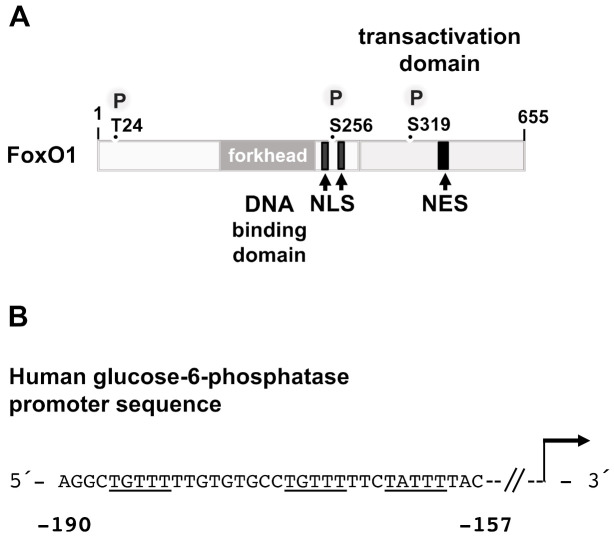
Modular structure and binding sites of FoxO1. (**A**) FoxO1 contains a DNA binding domain (forkhead) and a C-terminal transactivation domain. Nuclear localization signals (NLS), nuclear export signals (NES), and phosphorylation sites (P) are depicted. (**B**) FoxO1 binding sites within the human glucose-6-phosphatase gene promoter. The binding motifs are underlined. The sequence was obtained from GeneBank X96937.1.

The insulin-regulated transcription factor FoxO1 belongs to the group of winged-helix proteins. The “winged-helix” motif, also known as the Forkhead box, functions as a DNA binding domain, consisting of three α-helices and two large loops (termed “wings”). FoxO1 interacts as a monomer with the consensus core motif 5′-AAACA-3′ (or 5′-TGTTT-3′ on the opposite strand). The transactivation domain is located at the C-terminal end of the protein (Figure 7A). FoxO1 contains several nuclear localization signals as well as nuclear export signals, indicating that the nuclear-cytoplasmic shuttling is an important hallmark of the protein. FoxO1 is phosphorylated on several serine and threonine residues and acetylated by the histone acetyltransferases CBP/p300 on lysine residues K245, K247, and K265 [78].

Under resting conditions, i.e., when insulin levels are low, FoxO1 is found in the nucleus and transactivates genes that encode key enzymes of gluconeogenesis. Three potential FoxO1 binding sites have been identified within the proximal region of the glucose-6-phosphatase (G6Pase) gene promoter (Figure 7B). Mutations of these sites generate a promoter that is no longer regulated by FoxO1 and PKB/Akt [79]. A potential FoxO1 binding motif has also been identified in the PEPCK promoter. Expression of a dominant-negative mutant of FoxO1 in mouse hepatocytes prevented dexamethasone/cAMP-induced expression of both G6Pase and PEPCK [80], while expression of both G6Pase and PEPCK was significantly reduced in the liver of fasted transgenic mice that expressed only low levels of FoxO1 [81]. Likewise, G6Pase and PEPCK expression was reduced in the liver expressing a truncated, dominant-negative mutant of FoxO1 [82]. In contrast, G6Pase and PEPCK expression was increased in the liver of transgenic mice expressing a constitutively active mutant of FoxO1 [83]. In LIRKO mice, transgenic mice that lack the insulin receptor in the liver, a 6.9-fold increase in PEPCK mRNA concentration was observed. G6Pase expression was increased by 2.7-fold [84], reflecting the activation of FoxO1 due to the lack of insulin-induced Akt activation.

Experiments utilizing a constitutively active FoxO1 mutant revealed that FoxO1 stimulates glucose production in the liver via promoting gluconeogenesis. In contrast, glucose-consuming pathways such as glycolysis, the pentose phosphate pathway, and lipogenesis are attenuated [85]. FoxO1 exerts important effects on lipolysis by regulating adipose triacylglycerol lipase (ATGL) [83], the enzyme that catalyzes the first reaction in lipolysis.

Secretion of insulin activates, in insulin receptor-expressing cells, the protein kinase B/Akt which phosphorylates FoxO1 on residues T24, S256, and S329 (or T24, S253, S316 in *Mus musculus*). This results in an export of FoxO1 from the nucleus to the cytoplasm and a subsequent inhibition of gluconeogenic gene transcription. FoxO1 activity is extremely sensitive to insulin administration, leading to maximal phosphorylation within 30 s after insulin stimulation of hepatocytes [86]. Mutation of the phosphorylation sites to T24A, S256D, and S329A generates a constitutively active FoxO1 mutant that is no longer responsive to insulin and thus prevents insulin-induced inhibition of G6Pase and PEPCK expression [53]. FoxO1 executes its activity through the transcriptional coactivator peroxisome proliferative activated receptor-γ co-activator-1α (PGC-1α), which interacts with the amino-terminal-located binding site of the FoxO1 molecule. This interaction is strongly reduced by Akt-mediated phosphorylation of FoxO1. Accordingly, insulin administration decreased binding of PGC-1α and FoxO1 to the gluconeogenic genes encoding PEPCK and G6Pase in vivo [53]. In support of this, it has been shown that PGC-1α-induced expression of PEPCK and G6Pase was decreased by more than 95% in hepatocytes expressing low levels of FoxO1 [81], indicating that both PGC-1α and FoxO1 are essential for the activation of gluconeogenic genes. Together, insulin-induced activation of Akt promotes the sequestration of FoxO1 in the cytoplasm and further disrupts the interaction between FoxO1 and PCG-1α.

FoxO1 activity is not only regulated by phosphorylation, but also by acetylation and deacetylation involving acetyltransferases and deacetylases, which bind to the C-terminal activation domain of FoxO1. Acetylation, catalyzed by the acetyltransferases CBP and p300, attenuated the DNA binding activity of FoxO1 [87]. Knock-in mice containing a constitutively acetylated FoxO1 protein did not survive embryogenesis and resembled phenotypically FoxO1 knockout mice [88], indicating that acetylation serves as an “off” signal for FoxO1 activity. Deacetylation of FoxO1, catalyzed by nicotine-amide adenine dinucleotide-dependent deacetylase SIRT1 and by other deacetylases, potentiates FoxO1 transcriptional activity by promoting nuclear retention of FoxO1 [88]. FoxO1 contains a conserved C-terminal SIRT1 binding motif, encompassing the sequence LXXLL (amino acids 459–463). Mutation of this motif to AXXAA attenuated SIRT1 binding to FoxO1 and FoxO1 transcriptional activity [89]. In adipocytes, SIRT1 controls transcription of the adipose triglyceride lipase gene in particular and lipolysis in general by deacetylating FoxO1 [90].

Expression of a constitutively active mutant of FoxO1 has been shown to inhibit SREBP-1c expression and to further inhibit lipogenesis in the liver [85], indicating that the cytoplasmic retention of FoxO1 is essential for insulin-induced activation of SREBP-1c. In fact, expression of the FoxO1 mutant completely abolished insulin-induced transcription of a SREBP-1 promoter-controlled reporter gene. There is no FoxO1 binding site within the proximal SREBP-1c promoter. Rather, it has been suggested that FoxO1 inhibits the biological activity of SREBP-1c by preventing the assembly of the transcriptional activation complex of the SREBP-1 gene, by reducing LXR occupancy of the SREBP-1 promoter, and by interacting with the ubiquitously expressed transcription factor Sp1 [91,92].

## 4. Conclusions

This overview of insulin-responsive transcription factors shows that they are key regulators for executing many biological processes mediated by insulin. Insulin-responsive transcription factors are not limited to control of metabolic pathways. Rather, they affect glucose tolerance and insulin sensitivity of the entire organism. These proteins constitute a transcriptional network involving several transcription factors working together. The best example is the regulation of the fatty acid synthase gene by USF, SREBP, Liver X receptor, and ChREBP. Feed-forward activations have been identified, i.e., the regulation of the SREBP-1 gene by active SREBP-1c and LXR, or the activation of ChREBPβ transcription by ChREBPα and/or ChREBPβ. In contrast, there is a negative feedback loop involving the inhibition of SREBP-1c expression and transcriptional activity by FoxO1. This transcriptional network ensures that a biological function is controlled by multiple transcriptional regulators, as outlined for the regulation of lipogenic genes or genes encoding enzymes of the glycolytic pathway. The transcriptional network is supplemented by sharing a common coactivator such as PGC-1α (for FoxO1, LXR, and SREBP-1c). In addition, higher levels of regulation are executed by epigenetic regulators including acetyltransferases and deacetylase that are essential for the activity of insulin-responsive transcription factors. The counterpart of insulin-induced gene regulation is executed by PKA, which induces genes encoding for important enzymes of gluconeogenesis and lipolysis. Moreover, PKA directly inhibits the activity of the insulin-responsible transcription factors SREBP-1c, LXR, and ChREBP via phosphorylation.
